# How important are parents in the development of child anxiety and depression? A genomic analysis of parent-offspring trios in the Norwegian Mother Father and Child Cohort Study (MoBa)

**DOI:** 10.1186/s12916-020-01760-1

**Published:** 2020-10-27

**Authors:** Rosa Cheesman, Espen Moen Eilertsen, Yasmin I. Ahmadzadeh, Line C. Gjerde, Laurie J. Hannigan, Alexandra Havdahl, Alexander I. Young, Thalia C. Eley, Pål R. Njølstad, Per Magnus, Ole A. Andreassen, Eivind Ystrom, Tom A. McAdams

**Affiliations:** 1 Social Genetic & Developmental Psychiatry Centre, Institute of Psychiatry, Psychology & Neuroscience, King’s College London, London, UK; 2 Department of Mental Disorders, Norwegian Institute of Public Health, Oslo, Norway; 3 PROMENTA Research Center, Department of Psychology, University of Oslo, Oslo, Norway; 4 Nic Waals Institute at Lovisenberg Diaconal Hospital, Oslo, Norway; 5 MRC Integrative Epidemiology Unit, University of Bristol, Bristol, UK; 6 Center for Economic and Social Research, University of Southern California, Los Angeles, CA USA; 7 NIHR Maudsley Biomedical Research Centre, South London and Maudsley NHS Trust, London, UK; 8 Center of Diabetes Research, Department of Clinical Science, University of Bergen, Bergen, Norway; 9 Department of Pediatrics and Adolescents, Haukeland University Hospital, Bergen, Norway; 10 Centre for Fertility and Health, Norwegian Institute of Public Health, Oslo, Norway; 11 NORMENT Centre, Institute of Clinical Medicine, University of Oslo, Oslo, Norway; 12 Division of Mental Health and Addiction, Oslo University Hospital, Oslo, Norway; 13 School of Pharmacy, University of Oslo, Oslo, Norway

**Keywords:** Genomics, Environment, Genetic nurture, Anxiety, Depression, Children, MoBa

## Abstract

**Background:**

Many studies detect associations between parent behaviour and child symptoms of anxiety and depression. Despite knowledge that anxiety and depression are influenced by a complex interplay of genetic and environmental risk factors, most studies do not account for shared familial genetic risk. Quantitative genetic designs provide a means of controlling for shared genetics, but rely on observed putative exposure variables, and require data from highly specific family structures.

**Methods:**

The intergenerational genomic method, Relatedness Disequilibrium Regression (RDR), indexes environmental effects of parents on child traits using measured genotypes. RDR estimates how much the parent genome influences the child indirectly via the environment, over and above effects of genetic factors acting directly in the child. This ‘genetic nurture’ effect is agnostic to parent phenotype and captures unmeasured heritable parent behaviours. We applied RDR in a sample of 11,598 parent-offspring trios from the Norwegian Mother, Father and Child Cohort Study (MoBa) to estimate parental genetic nurture separately from direct child genetic effects on anxiety and depression symptoms at age 8. We tested for mediation of genetic nurture via maternal anxiety and depression symptoms. Results were compared to a complementary non-genomic pedigree model.

**Results:**

Parental genetic nurture explained 14% of the variance in depression symptoms at age 8. Subsequent analyses suggested that maternal anxiety and depression partially mediated this effect. The genetic nurture effect was mirrored by the finding of family environmental influence in our pedigree model. In contrast, variance in anxiety symptoms was not significantly influenced by common genetic variation in children or parents, despite a moderate pedigree heritability.

**Conclusions:**

Genomic methods like RDR represent new opportunities for genetically sensitive family research on complex human traits, which until now has been largely confined to adoption, twin and other pedigree designs. Our results are relevant to debates about the role of parents in the development of anxiety and depression in children, and possibly where to intervene to reduce problems.

## Background

Estimating how much parental behaviour influences children’s symptoms of anxiety and depression is important for understanding causes and designing interventions, but this is challenging. It cannot be assumed that associations between parental factors, such as control and hostility, and child outcomes, such as anxiety and depression [1–3], represent modifiable environmental effects. Genetic variation shared by parents and children may lead to spurious or inflated intergenerational associations. For instance, evidence suggests that the link between parental divorce and emotional problems in adult offspring is due to a common genetic component influencing parent and child traits, rather than an environmental effect of divorce [4].

Twin and pedigree studies help to separate genetic and environmental influences, and results suggest that environmental effects of parents on child traits, including anxiety and depression, are weak [5]. Specifically, the absence of shared environmental influence in twin studies points against effects of the parental behaviours that are useful modifiable exposures—those with systematic effects causing familial aggregation. Child anxiety symptoms are moderately genetically influenced, and the remaining variation appears to be non-shared environmental in origin [6]. For depression, shared environmental influences are more commonly detected, but are transient and decline across development [7]. However, twin and pedigree designs alone have limited utility for estimating parent effects. Shared environmental influence could be masked by interactions between genetic and shared environmental effects, which load onto the genetic component [8]. Moreover, shared environment estimates include effects of other sources of sibling resemblance (e.g. common friends), not only parents. Crucially, in the classical twin model, genes and environments are assumed to be uncorrelated, which can lead to biased estimates of shared environmental variance [9, 10].

Adoption and children of twins (CoT) designs provide stronger tests for environmental effects of parents on children than twin studies do, since they combine data on parents and children to correct for shared genetics. Intergenerational genetically sensitive research on child anxiety is scarce, but two studies suggest that parent-child associations for anxiety remain after controlling for genetic relatedness [11, 12]. The evidence base is larger for child depression. Numerous adoption and CoT studies indicate an environmental effect of parent depression above genetic confounding [13–16]. Harsh physical punishment and marital instability are also plausible environmental factors affecting child depression, which survive correction for genetic relatedness [17, 18]. Other putative influences on child depression, such as parent education [19], seem to be explained by shared genetics.

While adoption- and CoT-based parenting studies are valuable, their utility is limited in three ways. First, they focus on how specific measured parent phenotypes relate to child outcomes. This is useful for identifying or disqualifying specific causes. For instance, maternal depression after but not during pregnancy influences offspring emotional problems above genetic confounds [20, 21]. However, it is difficult to know a priori how best to identify and measure salient parent traits, and at what point in childhood to measure them. A second limitation is that the direction of effects is often not—or cannot be—assessed. Child-to-parent effects exist [11, 22–24], so where studies do not distinguish these from parent-to-child effects, associations cannot be interpreted as evidence for parental effects on child development. Third, reliance on highly specific samples (e.g. adoptees) may preclude large-scale data collection and reduce the generalisability of findings.

Novel intergenerational genomic designs present opportunities to address these limitations and extend insights from twin and adoption research. The Relatedness Disequilibrium Regression (RDR) method was introduced as a technique for estimating genetic influence without bias from the family environment [25]. A less emphasised attribute of this method is that RDR can estimate the environmental effects of parents. RDR measures the effect of parent behaviour indexed by their genome—termed ‘genetic nurture’ [26]. In RDR, estimates of genetic nurture capture all behaviours of both parents that are influenced by their common genetic variation, and that affects the child’s trait. Therefore, RDR bypasses the identification and measurement of specific parent traits. Unlike those estimated in adoption and CoT designs, the parent effect is global and a-temporal. Genetic nurture indexes any lasting parent effect from any parent phenotype, up to the time-point at which the child’s phenotype was measured. Additional advantages of RDR are that it allows child- versus parent-driven effects to be disentangled from each other and uses scalable population-based family samples rather than adoptive or twin families. A previous study detected genetic nurture for child depression using a similar method [27]. However, the analysis was underpowered, maternal and paternal effects were not modelled simultaneously, and mediating parent phenotypes were not explored.

Here, we apply RDR to 11,598 parent-offspring trios in the Norwegian Mother Father and Child Cohort Study (MoBa) to index environmental effects of parents on children’s anxiety and depression symptoms using the parental genome. Alongside parental genetic nurture, RDR estimates direct child genetic effects and the covariance between direct and nurturing effects. We use available maternal trait measures to test whether genetic nurture is mediated by self-reported maternal depression and anxiety. Additionally, we directly compare RDR results against traditional pedigree estimates, using 27,010 pairs of relatives (cousins, siblings, half-siblings and twins) from the child generation of MoBa.

## Methods

### Sample

The Norwegian Mother, Father and Child Cohort Study (MoBa [28]) is a prospective population-based pregnancy cohort study conducted by the Norwegian Institute of Public Health. Pregnant women were recruited from across Norway from 1999 to 2008. The women consented to initial participation in 41% of the pregnancies. The total cohort includes 114,500 children, 95,200 mothers and 75,200 fathers. To date, 98,110 individuals who are part of a trio (both parents and a child) from MoBa have been genotyped (Additional file 1: Table S1). As part of the Intergenerational Transmission of Risk (ITOR) subproject, MoBa has also been linked to Norwegian registry data containing pedigree and twin zygosity information. Version 11 of the quality-assured MoBa data files were used, released in 2018.

RDR and pedigree analyses required slightly different subsamples of MoBa families. For RDR, we used genotyped trios with available child anxiety and depression data. Siblings in the child generation were removed so that trios were independent families. For pedigree analyses, we retained all available cousins, siblings, half-siblings and twins in the child generation with anxiety and depression data, without requiring the presence of genotype data. Since only a subset of the families in MoBa was genotyped, all individuals included in genomic analyses were also within the sample used for pedigree analyses.

### Measures

Our two outcome variables are well-validated and reliable quantitative measures of childhood anxiety and depression symptoms at age 8. Anxiety was measured using the 5-item version of the Screen for Child Anxiety Related Disorders (SCARED) [29]. Depression was measured using the 13-item Short Moods and Feelings Questionnaire (SMFQ) [30]. Both questionnaires were rated by mothers using three-point Likert response scales. Phenotypes were regressed on child sex prior to analyses.

To test whether measured parent anxiety and depression symptoms explained any genetic nurture effects, we used mother-rated Hopkins Symptoms Checklist (SCL-8) [31]. The SCL-8 assesses self-report anxiety and depression symptoms experienced during the last 2 weeks. To obtain a reliable measure of maternal symptoms that children are consistently exposed to, we constructed a common factor of SCL-8 scores at five time-points: 15 weeks of pregnancy, 30 weeks of pregnancy, child age 6 months, 18 months, and 8 years. As has been demonstrated with childhood data [32], a stable factor composed of measurements at multiple time points better captures a heritable core trait than scores at a single time point. Applying this approach to assess maternal symptoms leads to a measure that captures stable exposure to maternal depression symptoms, rather than exposure associated with temporary fluctuations. Mediation of any genetic nurture effect by stable maternal anxiety and depression symptoms would be indicated by an observed change in the genetic nurture point estimate when using the factor score as a covariate.

### Genotype quality control

The current MoBa genomic dataset comprises imputed genetic data for 98,110 individuals (~ 32,000 parent-offspring trios), derived from nine batches of participants, who make up four study cohorts. Within each batch, parent and offspring genetic data were quality controlled separately. Quality control exclusion criteria for individuals were genotyping call rate < 95% or autosomal heterozygosity > 4 standard deviations from the sample mean. Quality control exclusion criteria for SNPs (single nucleotide polymorphisms) were ambiguous (A/T and C/G), genotyping call rate < 98%, minor allele frequency < 1%, or Hardy-Weinberg equilibrium *P* value < 1 × 10^–6^. Population stratification was assessed, using the HapMap phase 3 release 3 as a reference, by principal component analysis using EIGENSTRAT version 6.1.4. Visual inspection identified a homogenous population and individuals of non-European ancestries were removed based on principal component analysis of markers overlapping with available HapMap markers. The parent and offspring datasets were then merged into one dataset per genotyping batch, keeping only the SNPs that passed quality control in both datasets. Phasing was conducted using Shapeit 2 release 837 and the duoHMM approach was used to account for the pedigree structure. Imputation was conducted using the Haplotype reference consortium (HRC) release 1–1 as the genetic reference panel. The Sanger Imputation Server was used to perform the imputation with the Positional Burrows-Wheeler Transform (PBWT). The phasing and imputation were conducted separately for each genotyping batch. Additional file 1 Table S1 contains details of the numbers of SNPs and individuals in each batch. More detailed information about the cohorts, quality control and imputation can be found at https://github.com/folkehelseinstituttet/mobagen.

We conducted post-imputation quality control, selecting SNPs meeting the following criteria: high imputation confidence scores (INFO > 0.8 on average across batches), minor allele frequency > 0.05, Hardy-Weinberg equilibrium *p* > 1 × 10^–6^, non-multiallelic, and non-duplicated.

Before calculating relatedness matrices for RDR, we removed individuals who were not part of a complete genotyped trio, restricted to one child per family so that pairs of focal individuals did not share a home environment and pruned down to 451,442 variants in approximate linkage equilibrium using an *r*^2^ threshold of 0.5 (to reduce the size of the matrices and therefore the computational burden of the analyses).

After data management, a final sample of 25,828 genotyped parent-child trios was identified. Almost 11,600 of these trios also had child anxiety and depression symptom data.

### Statistical analyses

#### Genomic analysis of trios: relatedness disequilibrium regression (RDR)

The RDR method [25] allows the estimation of parent and child genetic effects on traits. This is achieved by extending a standard genomic method for estimating heritability—single-component GREML (Genomic-Relatedness based restricted Maximum-Likelihood) [33]—to include individuals’ parents. Standard GREML estimates the variance explained by common SNPs by comparing a matrix of pairwise genomic similarity for unrelated individuals across genotyped SNPs to a matrix of their pairwise phenotypic similarity, using a random-effects mixed linear model. Instead of using the random variation in genetic similarity among unrelated individuals, RDR estimates heritability by capitalising on the random variation in genetic similarity between pairs of individuals conditional on their parents’ genetic similarity, which arises through random segregation of alleles when gametes are formed, and is independent of environmental factors.

There are two versions of RDR: one uses identity-by-descent (IBD) relatedness, which distinguishes parts of the genome that are inherited from common ancestors, and the other uses common SNP-based relatedness. We used the SNP version since it has similar properties to the IBD version but has greater statistical power [25]. Rather than estimating a single genetic variance component, RDR estimates three. The first estimates the *direct* effect of children’s own genetic variation on their trait. This is independent of the effect of being reared by biological parents. Importantly, a direct genetic effect is only direct in the sense that it does not stem from another individual’s genotype. Notably, mechanisms by which individuals evoke and select environments based on their genotype are essential in how genes lead to phenotypes [34], and these are included in estimates of direct genetic influence.

The second variance component estimates the effect of parent genetics on the child trait, controlling for child genetic effects: ‘genetic nurture’. Any parent genetic effect over and above child-driven direct effects must be an *indirect* genetic effect, where parents’ genetics affect child traits by influencing parent behaviours and the rearing environment they provide. Notably, it is assumed that genetic nurture effects are from parents (not siblings) and that mating in the population is random. To the extent that these assumptions do not hold, the genetic nurture variance will be biased. Non-random mating would magnify the genetic nurture variance because it induces correlations between causal alleles across the genome, most importantly between transmitted and non-transmitted parts of the parental genomes [26].

The third component captures variance in the offspring phenotype attributable to covariance between the direct and nurturing genetic effects. This somewhat abstract variance component is easier to understand when considering the conditions for the estimate to be zero. Specifically, the direct-nurturing genetic covariance would not explain any phenotypic variation if only one generation contributes genetic effects to the child trait, or if different SNPs contribute to child and parent genetic influences, such that loci have only either direct or indirect effects. Covariance between direct and nurturing genetic effects can be thought of as a ‘passive gene-environment correlation’. This refers to a magnification of the environmental effect of genetically influenced parent behaviour, which happens because children passively inherit and are directly influenced by that same genetic material (see Additional file 1 Figure S1 for detail on this concept).

Finally, the residual component captures environmental effects on the trait of interest that are not correlated with measured parent genetic variation, the effects of variants not tagged by genotyped SNPs (e.g. rarer), and measurement error.

In practice, the variance components are estimated by regressing phenotypic resemblance on three genomic relatedness matrices simultaneously. The first is similar to the matrix used in standard GREML: the genome-wide genetic relatedness of the children in the sample. The second and third represent the genetic relatedness of the parents and the genetic covariance between children and parents.

Notably, the genotypes of mothers and fathers are combined to allow estimation of the effect of *both* parents. We calculated parental genotypes by summing the unnormalised maternal and paternal genotype matrices. We then standardised parental genotypes to have a mean zero and variance two. In an outbred random-mating population, the variance for the parental genotypes is twice that of the offspring genotype as it is the sum of maternal and paternal genotypes [25]. Notably, the summing of maternal and paternal genotypes contrasts to a similar model, M-GCTA [35], which does not involve paternal data but estimates the effects of the mother and child genomes and of their covariance.

All RDR analyses included 10 ancestry principal components and genotyping batch, both derived from the child generation, as covariates. Analyses were performed in the GCTA software. We used the --reml-no-constrain flag to allow components to take negative as well as positive values, given the theoretical and empirical evidence for negative covariance between direct and indirect genetic effects on complex traits. This can happen if a proportion of parental genetic variants associated with lower child trait scores are associated with higher child trait scores when present in the child genome. For example, studies have identified loci exert opposing maternal and fetal effects on human birth weight [36, 37].

To test whether any genetic nurture effect was partially explained by parent anxiety and depression symptoms, we re-ran the RDR models adding a measure of stable maternal anxiety and depression symptoms as a covariate. As mentioned above, this longitudinally-derived measure is preferable to time-specific measures as it captures a more reliable core trait that children are consistently exposed to. In addition, the measure maximises sample size and minimises bias in maternal reporting due to contemporaneous collection of maternal self- and child-report. We also tested the individual time-specific maternal measures as covariates.

To test whether any of the variance components were biased because some children were genotyped in a different batch to their parents, we re-ran the RDR models using only individuals who were genotyped as a complete trio (90% of the analysis sample).

To test the sensitivity of the genetic nurture estimate to a more stringent exclusion of relatives, we restricted all GRMs to relatedness < 0.1 and re-ran the RDR models.

Finally, to estimate standard SNP heritability, we ran single component GREML models using unrelated child genotypes. This is equivalent to running RDR with the genetic nurture and direct-nurturing genetic covariance components set to zero.

#### Classical pedigree modelling

To compare RDR results to a traditional quantitative genetic (non-genomic) design, we implemented a univariate pedigree model [38]. As in the classic twin design, this model allows estimation of genetic, shared environmental, and non-shared environmental (residual) influences on anxiety and depression symptoms at age 8. The model used phenotypic correlations among twins, siblings, half-siblings and cousins in the child generation, to derive estimates based on the following specifications: genetic correlations (assumed, not directly measured) are 1.00, 0.50, 0.25 and 0.125 for identical twins, non-identical twins/siblings, maternal half-siblings and cousins, respectively, and shared environmental correlations are 1.00 for all siblings and 0.00 for cousins. Sample sizes for pairs of relatives with available 8-year anxiety and depression data were 233 identical twins, 11,375 non-identical twins and siblings, 175 maternal half-siblings and 15,227 cousins, giving an overall sample of 27,010 pairs.

### Comparison of variance components from RDR and pedigree models

Before comparing RDR and pedigree results, it is essential to note the differences between variance components derived from the models. First, with respect to child genetic effects, RDR only captures additive effects tagged by measured common variants, but the pedigree genetic component also includes non-additive genetic effects, and the effects of rare variants not tagged by the common variants included on the SNP array. Second, regarding family environmental effects, the shared environment component in the pedigree model is broader than the genetic nurture estimate using RDR. The shared environment captures at least three sources of variance that genetic nurture does not parent behaviours not tagged by common SNPs, shared environments *beyond* parent behaviours, and effects of any correlation between the shared environment and genetic components (passive gene-environment correlation). Third, whereas pedigree and twin models assume passive gene-environment correlation is absent (i.e. that genetic and environmental effects are uncorrelated), RDR can directly estimate it as the covariance between genetic and environmental (genetic nurturing) effects.

Additional file 1 Figure S1 and its accompanying text explore overlaps and distinctions between the concepts of genetic nurture, passive gene-environment correlation and shared environment.

### Software

Genome-wide relatedness matrices were constructed using python, and RDR models were run using GCTA [33]. Pedigree analyses were conducted in R using the structural equation modelling package OpenMx [39].

## Results

### RDR model

**Fig. 1:**
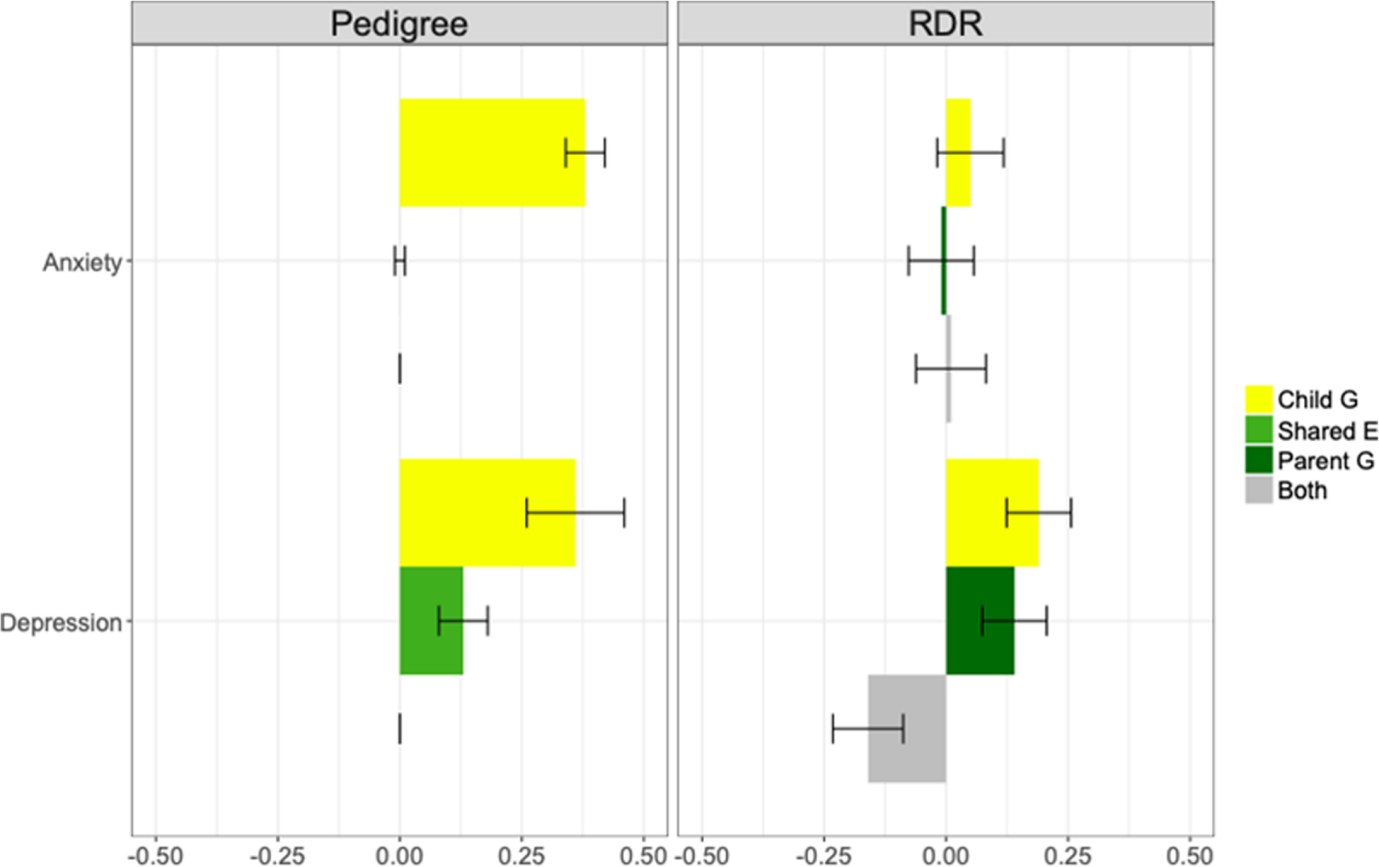
Genetic and environmental variance component estimates for anxiety and depression symptoms at age 8, from pedigree and RDR models. Coloured labels for the pedigree model refer to variance explained by genetic effects (i.e. pedigree heritability; yellow) and variance explained by shared environmental influences that increase resemblance among siblings in the same family (light green). Not shown: residual variance, which includes non-shared environmental effects and error. The effect of any covariance between genetic and shared environmental influences is not estimated in the pedigree model. Coloured labels for the RDR model refer to variance explained by direct child genetic effects (yellow), parent genetic effects (i.e. genetic nurture; dark green) and by covariance between the direct and nurturing genetic effects (‘Both’; grey). Not shown: residual variance affecting the phenotypes, including parent genetic effects not tagged by common SNPs, individual-specific environmental effects, chance factors and error. Sample sizes were ~ 27,000 pairs of related children for the pedigree model and ~ 11,600 genotyped parent-child trios for RDR. Bars = standard errors

Results from our RDR analyses using genomic data to disentangle child and parent genetic effects on anxiety and depression symptoms at age 8 are shown in Fig. 1. Direct effects of children’s own common genetic variation (yellow bars) explained 5% (se = 0.07) of the variance in anxiety symptoms and 19% (se = 0.07) of the variance in depressive symptoms. Genetic nurture (dark green bars) had a negligible influence on variation in child anxiety but explained 14% (se = 0.07) of the variance in child depression. For depression, the estimate of the phenotypic variance explained by covariance between direct and indirect genetic effects (i.e. passive gene-environment correlation) was negative (− 16%; se = 0.07; grey bars). This suggests that, on average, parental genetic variants associated with lower child depression symptoms are associated with higher child depression symptoms when present in the child genome.

See Additional file 1 Table S2 for full RDR results including sensitivity analyses exploring mediators, batch effects and the effect of constraining components to take positive values. In the mediation analysis, maternal anxiety and depression symptoms partially explained the genetic nurture effect on childhood depression. This manifested as the genetic nurture effect, but not the other variance components, being attenuated when including a covariate capturing stable maternal anxiety and depression symptoms (dropping from 14 (se = 0.07) to 5% (se = 0.07)). Most individual time-specific maternal measures also led to attenuation of the genetic nurture effect when included as covariates. To investigate how much the mediation was due to anxiety versus depression symptoms, we subsequently split the Hopkins measure (assessed when children were 8 years old) into its two sets of items measuring anxiety and depression. The genetic nurture effect (originally 14%; se = 0.07) attenuated to 5% (se = 0.07) when maternal depression symptoms were included as a covariate, and to 8% (se = 0.07) when maternal anxiety symptoms were included. Due to the small effect size difference and large overlapping errors, there is not sufficient evidence to suggest that maternal anxiety and depression play differing roles in explaining why the parental genome affects child depression. This is perhaps unsurprising given the strong phenotypic correlation between the anxiety and depression items (0.7).

Our results were robust to any effects of children being genotyped in a different batch to their parents. Variance components were unchanged after restricting the sample to trios who were genotyped together. Our results were also robust to more stringent exclusion of relatives: the genetic nurture variance for child depression remained 14% (se = 0.07) when all GRMs were computed using a relatedness cut-off of 0.1 (Additional file 1: Table S2).

Additional file 1 Figure S2 visualises the RDR results in a path diagram, with estimates of paths for direct and nurturing effects and of the correlation between them.

Single component GREML models (i.e. excluding parent genotype data from RDR) resulted in SNP heritabilities of 4% (se = 0.05) and 10% (se = 0.05) for anxiety and depression, respectively. These results are equivalent to the following calculation based on the RDR estimates: direct genetic effect + 1/2 genetic nurturing effect + covariance effect [25]. The genetic nurture effect is halved because only the genetic material transmitted from parents is relevant. For example, the SNP heritability of child depression (10%) equates to 19 + (14/2) − 16 (from the main RDR results). This indicates that in the presence of negative direct-nurturing genetic covariance, SNP heritability estimates based on single-component GREML will be underestimated.

### Pedigree model

Our key finding of genetic nurture for child depression but not anxiety was supported by our pedigree analysis (Fig. 1). Shared environmental influences contributed 13% (se = 0.05) of the variation in depression, but we observed no robust evidence of shared environmental effects on anxiety. The pedigree heritability estimates were similar for anxiety and depression (~ 37%). This contrasts to the pattern of heritability estimates from RDR, in which child genetic effects explained more variance in depression than anxiety (19% versus 5%, respectively). See Additional file 1 Figure S3 for a path diagram version of the pedigree results, plus model fit statistics.

## Discussion

We used genomic data from parent-offspring trios to estimate indirect parent genetic effects (genetic nurture) separately from direct child genetic effects on anxiety and depression symptoms at age 8. Results from the RDR model suggested that depressive symptoms were influenced by both genetic nurture and direct genetic effects. Genetic nurture is an environmentally mediated effect of genetic origin, indexing behaviours of both parents that are influenced by their common genetic variation and that influence child depression, over and above direct child genetic effects. We found that the genetic nurture effect was partially mediated via an observed measure of maternal anxiety and depression, which explained 64% of the original genetic nurture effect (14%; se = 0.07)). In contrast, individual differences in anxiety symptoms were not significantly influenced by parental genetic nurture, and direct effects of common genetic variants explained just 5% (se = 0.07) of the phenotypic variance.

### Genetic nurture for child depression symptoms

Our genomic evidence of an environmental effect of parents on child depression, but not anxiety, was supported by pedigree-based evidence for shared environmental influence on depression, but not anxiety. This aligns well with findings from epidemiological and twin studies. Unlike anxiety, depressive symptoms are uncommon in pre-pubertal children, but when they do occur, are typically a response to psychosocial risks, such as perinatal issues, abuse and bullying [40, 41]. Twin data also suggest a greater role for family environmental factors in child depression than anxiety. In one twin study, shared environmental effects contributed 18% of the variance in depressive symptoms in middle childhood, but none for anxiety [32]. The use of RDR adds to our knowledge of parent effects from pedigree methods, because genetic nurture is simultaneously specific to parents and global. In contrast, estimates of parental influence from twin studies (i.e. the shared environment) are not specific to parents, and those from the children of twins (CoT) method rely on phenotypic measurement of specific parent traits.

### Mediation by maternal anxiety and depression

Our analysis adjusting for a longitudinal measure of stable maternal anxiety and depression suggested that these symptoms partially mediate the genetic nurture effect on childhood depression. This is compatible with adoption- and CoT-based evidence for phenotypic effects of maternal depression [16]. This environmental influence could reflect a learning mechanism directly related to maternal anxiety and depression symptoms or could be mediated through secondary parent behaviours such as disorganised home environments [16, 42]. Indeed, the large mediation effect could reflect that our maternal measure captures many salient aspects of the environment of the child that occur with stable maternal anxiety and depression symptoms, such as paternal mental health problems, marital discord and social disadvantage [43]. Therefore, although the strong maternal mediation effect appears to leave little room for father effects, maternal symptoms could be partially indexing the effects of paternal depression, which has been linked to depression symptoms in young people [52].

### Negative covariance between direct and nurturing genetic effects on child depression

This negative component suggests that genetic variation that is associated with higher depression risk when present in children, has an opposing effect (*reducing* child depression risk) when present in the parents. On the face of it, this result is difficult to explain and we suggest that it should be interpreted with caution. Notably, negative covariance components have been reported in previous RDR analyses of multiple phenotypes including creatinine levels and age of the first child in men [25], and in a Maternal-GCTA analysis of gestational weight gain [37]. Negative direct-indirect covariances are frequently detected in studies of parental care in animals [44]. One hypothesis regarding our finding is that genetic variants that influence mothers to identify depressive symptoms in their children could be linked to emotional sensitivity and may also be associated with parent behaviours that reduce offspring depression symptoms.

### Pedigree and genomic heritabilities

For child anxiety, the pedigree heritability estimate was moderate, despite the small genomic signal from RDR. This is compatible with findings from other samples [45]. However, it is surprising that the SNP-based effects are lower for anxiety than depression, given that they are similar traits, with similar pedigree heritability estimates. It could be that anxiety, more than depression, is influenced by non-additive or rare genetic effects, or by gene-by-shared environment interactions. These effects are captured in pedigree but not RDR heritability estimates, since the latter only considers additive genetic effects tagged by common SNPs, and does not compare children who share a family environment.

### Limitations

We acknowledge potential sources of bias affecting the RDR results. First, although supportive of the notion of parental influence on child depressive symptoms, the genetic nurture estimate may also capture residual population stratification, assortative mating and indirect genetic effects from siblings [26]. Population stratification is unlikely to explain a large proportion of the genetic nurture effect, since we adjusted for principal components. Assortative mating is also unlikely to explain our main finding. Non-random mating with respect to depression and anxiety is usually found to be moderate or absent and results in negligible bias of the shared environmental component in twin studies [46, 47]. More research is needed to evaluate indirect genetic effects of siblings on children’s emotional symptoms, especially given that sibling bullying predicts depression in early adulthood [48].

Second, the genetic nurture effect, and the mediation effect, could be partly generated by the use of maternal ratings of child outcomes. Maternal reports of child depression symptoms may reflect their own anxiety and depression symptoms. As a result, an apparent parental genetic nurture effect may in fact be a direct genetic effect on parents’ own traits. This phenomenon has been evidenced in developmental twin studies, in which rater bias may cause shared environmental variance to be overestimated [49]. However, if rater bias explained the environmental effect for depression, then we might expect to see some evidence for this with anxiety too. The lack of a genetic nurture effect for anxiety in our RDR and pedigree models suggests that maternal report is not entirely reflective of maternal phenotype. Furthermore, our use of a longitudinal measure of maternal symptoms reduces the chance that the mediation effect is inflated by mothers reporting contemporaneously on maternal and child symptoms. Measurement issues will be clarified with the upcoming availability of child, teacher and clinician reports in MoBa.

Third, selective participation, genotyping and attrition might have reduced coverage of families experiencing more severe anxiety and depression symptoms. Depression-linked attrition has been demonstrated in the Norwegian Twin Panel [50]. Stronger parent effects might occur at the tails of the distribution of child anxiety and depression. Future linkage of MoBa with Norwegian registry data will help to investigate and control for participation biases affecting the genotyped trios.

### Future directions

Future studies could seek to identify phenotypes other than maternal anxiety and depression symptoms that explain why the parent genome independently influences child depression. To aid the discovery of specific mechanisms, it will be important to jointly estimate maternal and paternal genetic nurture [51], the relationship between these effects and their respective mediating phenotypes. Ultimately, integrating fine-grained information on parenting styles such as warmth and hostility will yield the most precise insights. Since common genetic variants only capture a subset of the genetic component of complex traits involved in parenting, our observed genetic nurture effect on childhood depression is likely to be larger than we estimate here. This implies substantial scope for identifying heritable parent phenotypes with effects on child depression symptoms. However, individual parenting phenotypes may have small effect sizes and are usually only modestly heritable (~ 20%) [53]. Researchers should therefore continue to explore wider social factors that might affect parents, and in turn, childhood anxiety and depression. Mediators of genetic nurture might depend on socioeconomic context. For example, poverty increases the magnitude of the effect of maternal depression on diverse child outcomes [54]. In the future, RDR could be adapted to allow moderation effects to be tested.

The RDR method could be broadened to address multivariate and longitudinal questions, as has been done with twin designs [55]. For example, does genetic nurture persistently influence depressive symptoms across development? Twin research suggests that the effects of parents will be stronger earlier in life [56–58]. This is partly because with increasing age less time is spent in the family environment, and individuals have greater capacity to choose their experiences and express their genetic predispositions. Genomic tools could be used to strengthen and develop findings from traditional designs.

## Conclusions

Our genomic approach strengthens the evidence base regarding parent effects on child emotional development, by directly accounting for shared genetics, and by indexing the child’s environment using the parental genome. Quantifying the contribution of parents is crucial for the understanding of how anxiety and depression develop in childhood, and potentially how to target modifiable causes. Our results are supportive of parent effects on depressive symptoms at age 8. The genetic nurture effect on depressive symptoms was partially mediated by maternal anxiety and depression. Though the effect size is modest, this suggests that efforts to alleviate maternal anxiety and depression could help to prevent child depression.

## Supplementary information

**Additional file 1 : Table S1.** MoBa genotyping and imputation information. **Table S2**. Full RDR results. **Figure S1**. Comparison of related concepts: passive gene-environment correlation, genetic nurture, and the shared environment. **Figure S2**. Simplified path diagram for RDR results. **Figure S3**. Simplified path diagram for pedigree results.

**Additional file 2.** Code for running the Relatedness Disequilibrium Regression model.

## Data Availability

MoBa data are available to individuals who obtain the necessary permissions from the data access committee. Analysis code for the Relatedness Disequilibrium Regression model is provided in Additional file 2.

## Abbreviations

MoBa: Norwegian Mother Father and Child Cohort Study
SNP: Single nucleotide polymorphism
RDR: Relatedness Disequilibrium Regression
