# Supplementary material for: How important are parents in the development of child anxiety and depression? A genomic analysis of parent-offspring trios in the Norwegian Mother Father and Child Cohort Study (MoBa)
Source: BMC Med. 2020 Oct 27;18:284. doi: 10.1186/s12916-020-01760-1 (PMC7590735; doi:10.1186/s12916-020-01760-1)

Additional File 1

Contents:

*Table S1. MoBa genotyping and imputation information*

*Table S2. Full RDR results*

*Figure S1. Comparison of related concepts: passive gene-environment correlation, genetic nurture, and the shared environment*

*Figure S2. Simplified path diagram for RDR results*

*Figure S3. Simplified path diagram for pedigree results*

*Table S1.* MoBa genotyping and imputation information

| **Cohort** | **Genotyping chip** | **Genotyping center** | **Cohort design** | **N SNPs** | | | **N individuals** | | |
| --- | --- | --- | --- | --- | --- | --- | --- | --- | --- |
|  |  |  |  | **Genotyped** | **Used for imputation** | **Imputed** | **Sent** | **Genotyped** | **Imputed** |
| Harvest | Illumina HumanCoreExome12v1.1 | Genomics core facility, Trondheim, Norway | Trios | 542,585 | 384,406 | 39,975,206 | 20,668 | 1,692 | 20,231 |
|  |  |  |  | 542,585 |  |  |  | 18,972 |  |
|  | Illumina HumanCoreExome24v1.0 |  |  | 547,644 | 395,736 | 39,963,876 | 12,874 | 12,874 | 12,757 |
| Rotterdam | Illumina Global Screening Array MD v.1.0 | ERASMUS MC, Rotterdam, Netherlands |  | 692,367 | 567,903 | 39,791,709 | 27,000 | 17,949 | 17,742 |
|  | Illumina Global Screening Array MD v.1.0 |  |  | 692,338 | 571,759 | 39,787,853 |  | 9,041 | 8,959 |
| Norment | Illumina Global Screening Array MD v.01.0 + 50k custom content (OmniExpress overlap) | deCODE Genetics, Rekjavik, Iceland | Trios | 693,143 | 588,807 | 39,770,805 | 9,841 | 9,632 | 9,628 |
|  | Illumina HumanOmniExpress-24v1.0 |  | Unrelated parents | 708,882 | 665,648 | 39,693,964 | 6,040 | 2,976 | 2,851 |
|  | Illumina HumanOmniExpress-24v1.0 |  |  | 710,146 | 667,978 | 39,691,634 |  | 2,983 | 2,983 |
|  | Illumina InfiniumOmniExpress-24v1.2 |  | Trios | 712,628 | 683,577 | 39,676,035 | 19,611 | 17,730 | 17,730 |
| ADHD | Illumina InfiniumOmniExpress-24v1.2 |  | Case trios and singleton controls | 713,599 | 670,860 | 39,688,752 | 5,790 | 5,410 | 5,229 |
| **MoBa Genetics Release 1** |  |  |  |  |  |  | **101,824** | **99,259** | **98,110** |

*Table S2.* Full RDR results*.* Note: Vg= direct effect of child genetics; Ve~g = genetic nurture i.e. variance in child’s environment explained by parent genetics; Cg,e = variance explained by covariance between direct and nurturing genetic effects; SE=standard error; sex resid.=outcome residualised for sex; mat anx/dep=maternal anxiety and depression symptoms; W/M/Y=weeks months years of the child’s life. Key results from the main text are in bold.

| Phenotype | Covariates | Vg | Ve~g | Cg,e | SE_Vg | SE_Ve~g | SE_Cg,e | N trios | logL |
| --- | --- | --- | --- | --- | --- | --- | --- | --- | --- |
| Anxiety (sex resid.) | NA | 0.05 | 0 | 0.03 | 0.07 | 0.07 | 0.07 | 11598 | -5801.71 |
| **Anxiety (sex resid.)** | **10 PCs, batch** | **0.05** | **-0.01** | **0.01** | **0.07** | **0.07** | **0.07** | **11598** | **-5793.05** |
| Anxiety (sex resid.) | 10 PCs, batch, longitudinal mat anx/dep | 0.01 | -0.02 | 0.05 | 0.07 | 0.07 | 0.07 | 11566 | -5660.32 |
| Anxiety (sex resid.) | 10 PCs, batch, mat anx/dep at 15W | 0.03 | 0.02 | 0.02 | 0.07 | 0.07 | 0.07 | 11409 | -5658.27 |
| Anxiety (sex resid.) | 10 PCs, batch, mat anx/dep at 30W | 0.04 | -0.01 | 0.03 | 0.07 | 0.07 | 0.07 | 11350 | -5646.90 |
| Anxiety (sex resid.) | 10 PCs, batch, mat anx/dep at 6M | 0.03 | -0.03 | 0.05 | 0.07 | 0.07 | 0.07 | 11261 | -5598.43 |
| Anxiety (sex resid.) | 10 PCs, batch, mat anx/dep at 18M | 0.04 | -0.03 | 0.03 | 0.07 | 0.07 | 0.08 | 10571 | -5251.53 |
| Anxiety (sex resid.) | 10 PCs, batch, mat anx/dep at 3Y | -0.01 | -0.08 | 0.07 | 0.08 | 0.09 | 0.09 | 9607 | -4667.34 |
| Anxiety (sex resid.) | 10 PCs, batch, mat anx/dep at 8Y | -0.01 | -0.05 | 0.09 | 0.07 | 0.07 | 0.08 | 11409 | -5587.07 |
| Anxiety (sex resid.) - only trios genotyped on  the same batch | 10 PCs, batch | 0.07 | -0.02 | 0.04 | 0.08 | 0.08 | 0.08 | 10378 | -5184.81 |
| Anxiety (sex resid.), components constrained  >0 | 10 PCs, batch | 0.05 | 0 | 0.01 | 0.07 | 0.07 | 0.07 | 11598 | -5793.05 |
| Anxiety (sex resid.) - all GRMs restricted to  relatedness <0.1 | 10 PCs, batch | 0.001 | -0.05 | 0.07 | 0.08 | 0.08 | 0.09 | 10339 | -5170.92 |
| Depression (sex resid.) | NA | 0.16 | 0.13 | -0.1 | 0.07 | 0.07 | 0.07 | 11584 | -5789.02 |
| **Depression (sex resid.)** | **10 PCs, batch** | **0.19** | **0.14** | **-0.16** | **0.07** | **0.07** | **0.07** | **11584** | **-5657.74** |
| **Depression (sex resid.)** | **10 PCs, batch, longitudinal mat anx/dep** | **0.17** | **0.05** | **-0.11** | **0.07** | **0.07** | **0.07** | **11552** | -5293.88 |
| Depression (sex resid.) | 10 PCs, batch, mat anx/dep at 15W | 0.21 | 0.09 | -0.13 | 0.07 | 0.07 | 0.07 | 11395 | -5382.96 |
| Depression (sex resid.) | 10 PCs, batch, mat anx/dep at 30W | 0.15 | 0.06 | -0.09 | 0.07 | 0.07 | 0.07 | 11335 | -5344.26 |
| Depression (sex resid.) | 10 PCs, batch, mat anx/dep at 6M | 0.16 | 0.07 | -0.11 | 0.07 | 0.07 | 0.07 | 11247 | -5332.71 |
| Depression (sex resid.) | 10 PCs, batch, mat anx/dep at 18M | 0.2 | 0.13 | -0.16 | 0.07 | 0.07 | 0.08 | 10559 | -5030.55 |
| Depression (sex resid.) | 10 PCs, batch, mat anx/dep at 3Y | 0.17 | 0.01 | -0.09 | 0.08 | 0.08 | 0.09 | 9595 | -4431.16 |
| Depression (sex resid.) | 10 PCs, batch, mat anx/dep at 8Y | 0.15 | 0.05 | -0.12 | 0.07 | 0.07 | 0.07 | 11390 | -5126.78 |
| Depression (sex resid.) | 10 PCs, batch, mat anx at 8Y | 0.17 | 0.08 | -0.14 | 0.07 | 0.07 | 0.07 | 11390 | -5292.45 |
| Depression (sex resid.) | 10 PCs, batch, mat dep at 8Y | 0.14 | 0.05 | -0.12 | 0.07 | 0.07 | 0.07 | 11390 | -5104.96 |
| Depression (sex resid.) - only trios genotyped on the same batch | 10 PCs, batch | 0.19 | 0.12 | -0.18 | 0.07 | 0.07 | 0.08 | 10364 | -4927.15 |
| Depression (sex resid.), components constrained  >0 | 10 PCs, batch | 0.14 | 0.09 | 0 | 0.07 | 0.07 | 0.07 | 11584 | -5658.37 |
| Depression (sex resid.) - all GRMs restricted to  relatedness <0.1 | 10 PCs, batch | 0.23 | 0.14 | -0.17 | 0.07 | 0.07 | 0.08 | 10327 | -5104.67 |

*Figure S1.* Comparison of related concepts: passive gene-environment correlation, genetic nurture, and the shared environment*.* Note: G=genetic, P=phenotype, Par=parent.

The term ‘genetic nurture’ was coined recently (Kong et al. 2018), but overlaps somewhat with existing concepts from quantitative genetics: passive gene-environment correlation and shared environment. We now discuss the similarities and differences between these concepts with reference to Figure S1. In Figure S1, the child phenotype is influenced directly by genes present in the child (red). Since children are genetically related to their parents, direct genetic effects become correlated (green) with genetic effects affecting children *indirectly* via parental behaviour (blue).


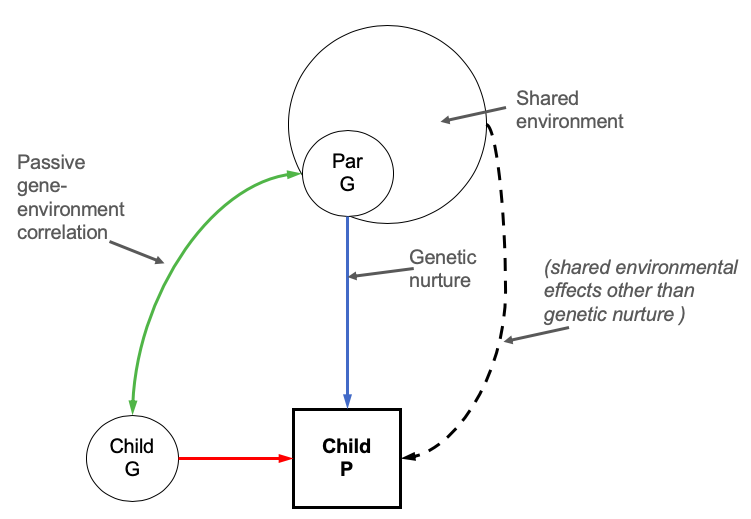


Genetic nurture and passive gene-environment correlation both refer to the intertwining of genetic and environmental effects on a child phenotype that occurs because parental behaviour is heritable. ‘Genetic nurture’, estimated using intergenerational genomic data, describes the indirect effect of the parent genome ***over and above* the effects of genes shared with the child.** This can be assessed by estimating effects of non-transmitted alleles (Kong et al. 2018; Bates et al. 2018), or by estimating the independent effect of parental genotypes in a multiple regression controlling for effects of child genotypes (i.e. the blue path in SF1 is estimated, adjusting for the green and red paths). In contrast, ‘passive gene-environment correlation’ refers to a correlation between child genetic effects and environmental effects, including parental genetic nurture effects. Passive gene-environment correlation arises due to **shared genes** between parents and children (green double-headed arrow). In the RDR model, this is estimated as the covariance between the direct and nurturing genetic effects. This differs slightly from how the term is sometimes used in twin studies to stress shared genes independently affecting child and parent phenotypes. There, the parent genetic effect is on a measured parent phenotype, and does not necessarily in turn affect the child.

Since genetic nurture represents an **environmentally-mediated** genetic effect of parents, rather than an effect arising from the **correlation** between genetic and environmental effects, it may be conceptually closer to estimates of the shared environmental variance in the classical twin design than to passive gene-environment correlation (note that genetic nurture and passive gene-environment correlation are likely to both load onto the shared environment component in twin models). Figure S1 depicts how the shared environment is highly overlapping with genetic nurture. Both constructs capture main effects of the family environment, which are uncorrelated with child-specific genetic effects, and therefore do not differ among siblings. Genetic nurture only includes parent effects captured by their common genetic variation, whereas shared environmental influence (indicated by the dashed arrow) also includes any non-genetic effect making siblings similar. However, it cannot be assumed that genetic nurture effects are necessarily a subset of the shared environment variance estimated in a pedigree design. Genetic nurture may also share properties with the genetic component in pedigree models, because it would also increase resemblance among cousins and half siblings when they are included in the model (the shared environment is typically defined as one that is shared by siblings living together so would not make e.g. cousins similar).

## *Figure S2*. Simplified path diagram for RDR results. Note: Bold=focal phenotype, G=genetic, Par=parent. Red and blue values show the direct child genetic and parental genetic nurture path estimates, respectively. The green value for depression represents the correlation between direct and nurturing genetic effects. This was estimated by dividing the covariance component [-0.16] by two times the square root of the product of the variance components [(2*√(0.19*0.14)]. The correlation could not be computed for anxiety, due to the negative genetic nurture path estimate. Not shown: residual effects on the phenotypes, including parent genetic effects not tagged by common SNPs, individual-specific environmental effects, chance factors and error.

##
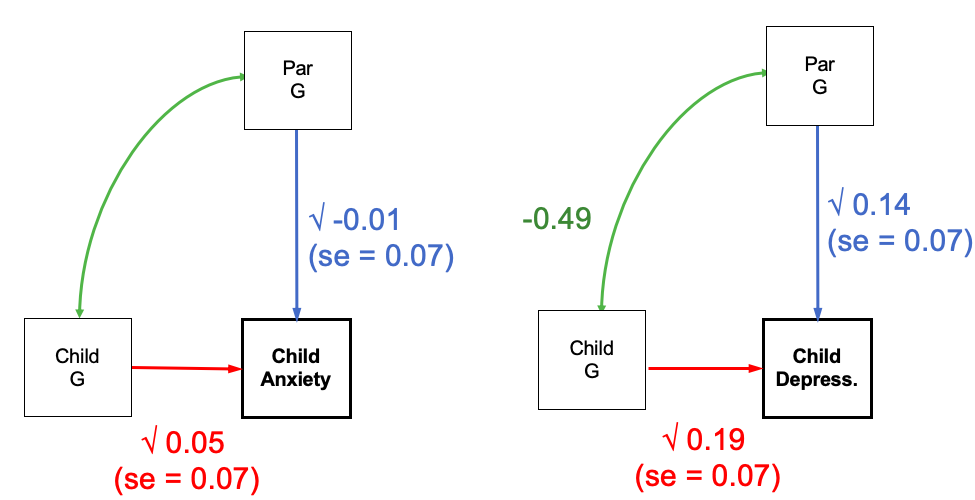


##

*Figure S3.* Simplified path diagram for pedigree results. Note: Bold=focal phenotype, square=observed, circle=latent, G=genetic, C= shared environment. Not shown: residual (includes non-shared environmental effects and error). The correlation between genetic and shared environmental influences is not estimated in this model. The full ACE models fitted the data well: TLI>.99 and RMSEA <.05 for both anxiety and depression; -2LL=56763.24 for anxiety and 55376.32 for depression


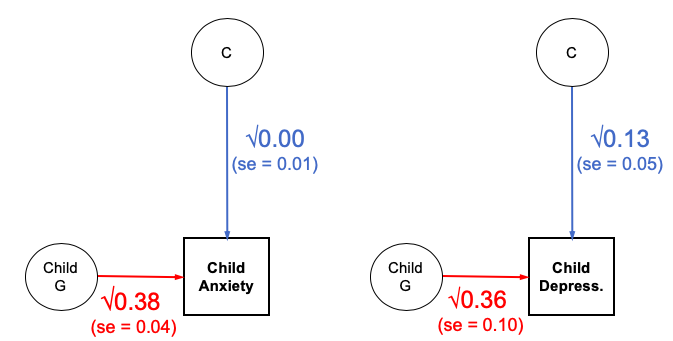

Supplement: Supplementary file 1 — Additional file 1 : Table S1. MoBa genotyping and imputation information. Table S2. Full RDR results. Figure S1. Comparison of related concepts: passive gene-environment correlation, genetic nurture, and the shared environment. Figure S2. Simplified path diagram for RDR results. Figure S3. Simplified path diagram for pedigree results. [file 12916_2020_1760_MOESM1_ESM.docx]
